# Adaption and validation of simplified Chinese version of the Low Back Pain Knowledge questionnaire (sC-LKQ)

**DOI:** 10.3389/fpubh.2023.1232700

**Published:** 2023-09-29

**Authors:** Zhe Wang, Yinyao Xie, Olívia Dózsa-Juhász, Alexandra Makai, Melinda Járomi

**Affiliations:** ^1^Doctoral School of Health Sciences, Faculty of Health Sciences, University of Pécs, Pécs, Hungary; ^2^Xi’an Honghui Hospital, Xi’an, China; ^3^Institute of Physiotherapy and Sport Science, Faculty of Health Sciences, University of Pécs, Pécs, Hungary

**Keywords:** low back pain, knowledge, questionnaire, reliability, validity

## Abstract

**Introduction:**

Low back pain (LBP) knowledge questionnaire (LKQ) was developed by a Brazilian research group in 2009. It has been cross-culturally adapted to many languages with good reliability and validity. This work aimed to translate and validate the LKQ into a simplified Chinese version and to evaluate the self-efficacy in LBP among Chinese participants from China and Hungary.

**Methods:**

A total of 431 people participated in this research, which lasted from September 2021 to June 2022 and was conducted on the Credamo online platform. The simplified Chinese LKQ (sC-LKQ) was generated through translation and cross-cultural adaptation guidelines. The participants were selected to fill out demographic questions, the sC-LKQ, and the Roland-Morris Disability Questionnaire (RMDQ). The reliability and validity of the data were evaluated using SPSS 28.0.

**Results:**

The sC-LKQ showed good internal consistency (Cronbach’s alpha was 0.79), and the intraclass correlation value was 0.85. There were five components in the questionnaire with good construct validity. The scores of RMDQ had negatively correlated with sC-LKQ.

**Conclusion:**

In the Chinese population, the sC-LKQ demonstrated excellent psychometric qualities and could be used to evaluate self-efficacy in clinical practice and research.

## 1. Introduction

Low back pain (LBP) has been one of the major factors affecting years lived with disability globally for the past three decades and carries a large public health burden (1–3). Understanding the disease-specific aspects of LBP is crucial for both prevention and treatment of spinal diseases (4, 5). It allows for targeted efforts to reduce risk, enables early intervention, supports the development of personalized treatment plans, and contributes to ongoing research and innovation in the field of LBP management (6, 7). Some researchers have found a link between disease-specific knowledge with effective prevention and rehabilitation (8, 9). Therefore, knowledge of specific diseases can be developed through educational programs (e.g., spinal school programs) and measured through knowledge questionnaires. Knowledge about the prevention and rehabilitation of spinal disorders can be assessed with the Low Back Pain Knowledge Questionnaire (LKQ) originally developed by Maciel et al. in 2009 (10). It was translated and validated into the Arabic (2017) and Hungarian (2019) languages (11, 12).

In China, the prevalence of LBP is increasing because of the higher mean age and life expectancy of the population (13, 14). Spinal pain is anticipated to worsen the public health burden with population aging (15). The prevalence of LBP does not have a specific population pattern; it shows in different occupations (16, 17) and has even become one of the health concerns of adolescents (18). It is important to improve knowledge of LBP disorders. The simplified Chinese version of the LKQ (sC-LKQ) has not been validated, and clinicians do not have an efficient tool to assess LBP knowledge. This study aimed to translate and validate the original LKQ into simplified Chinese and also explored the characteristics among the participants.

## 2. Methods and materials

### 2.1. Participants

The factor analysis requires a sample size of five to 10 times the number of entries and takes into account a sample inefficiency of 10% (19). The sample size for the current validation of the sC-LKQ was at least 176 for the 16 items. There were 431 participants recruited through hospital outpatient clinics and online social platforms to participate in the cross-sectional quantitative study in China and Hungary between September 2021 and June 2022. The number of participants who met the criteria for conducting the health questionnaire (20). The inclusion criteria were as follows: (1) older than the age of 18; (2) native Chinese speakers living in China or Hungary. The Exclusion criteria were as follows: (1) a history of tumors, current low back infection, and other conditions specifically linked to pain; (2) inability to complete the questionnaire independently; and (3) learning difficulties or dyslexia.

Of these, three participants were excluded because of improper completion of the questionnaire. Finally, we ultimately included data from 428 participants. Data were collected online using the Credamo questionnaire platform. The study was approved by the Local Ethics Committee of Chengdu Sports University Hospital No.2020002 and the Institutional Review Board of the Regional Research Committee of Clinical Center at the University of Pécs No.8342-PTE 2020. All participants signed the informed consent form.

All the participants were divided into six groups:

Group 1: Healthy people without health sciences or medical education background in China.

Group 2: Healthy people with health sciences or medical education backgrounds in China.

Group 3: LBP patients who received ambulatory treatment in China and had LBP confirmed by imaging examination.

Group 4: people who had LBP history within 1 year in China.

Group 5: Chinese people living in Hungary with health sciences or medical education backgrounds.

Group 6: Chinese people living in Hungary without health sciences or medical education backgrounds.

Sixteen participants were chosen at random from the entire sample to test the repeatability of the instruments.

### 2.2. LKQ translation and cross-cultural adaptation

**Figure 1:**
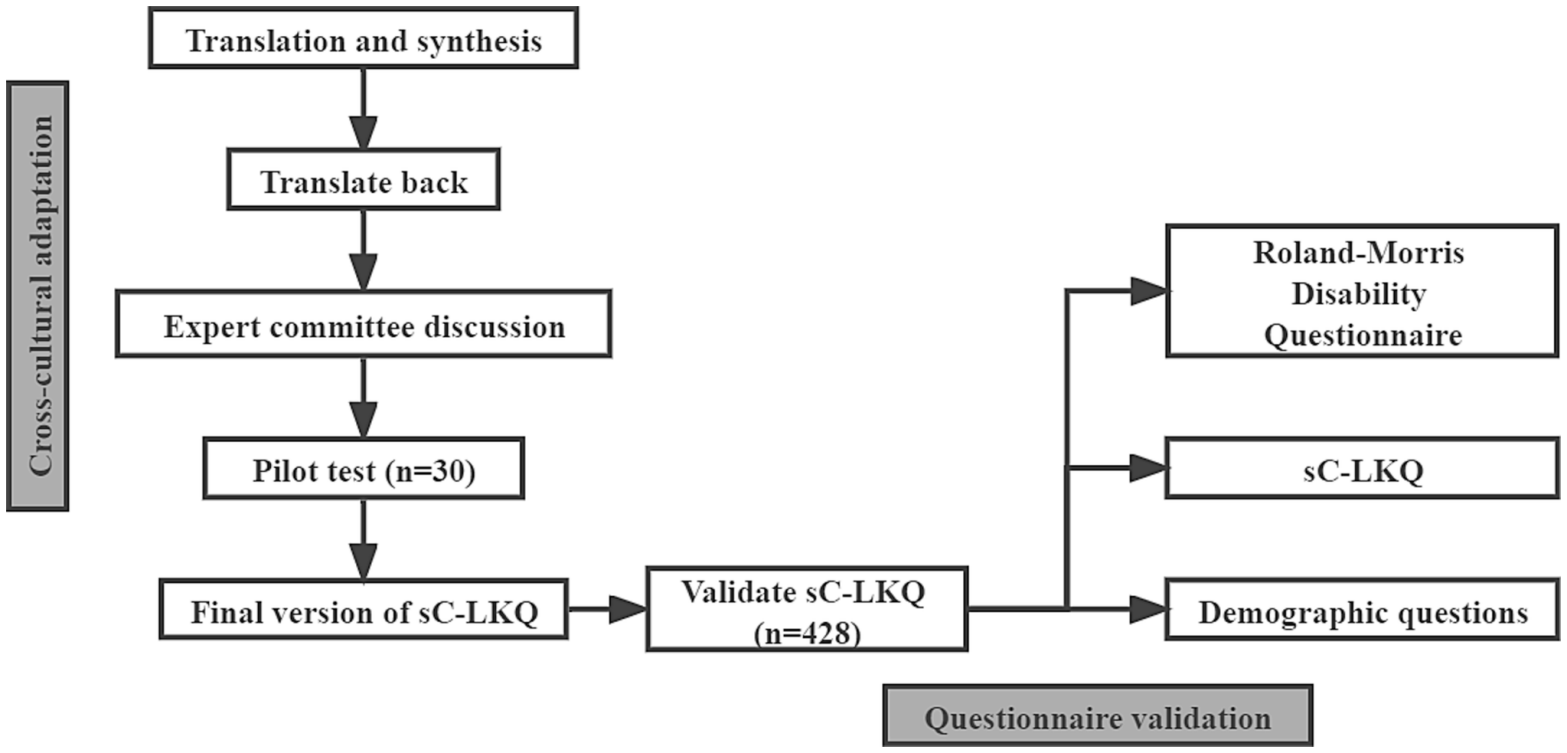
Flowchart of cross-cultural adaption and validation of sC-LKQ.

The LKQ translation into a simplified Chinese version was authorized and permitted by inventor Marciel. The whole translation and validation process was performed according to Beaton’s guidelines for the process of cross-cultural adaptation of self-report (21). It includes six steps: translation, synthesis, back translation, getting in common through expert committee, testing of the prefinal version, and obtaining the final version (Figure 1).

Two independent experts with a multilingual medical educational background translated the LKQ English version into a simplified Chinese version. Only one of them was a physiotherapist who knew the details of the LKQ. Based on the two translated questionnaires, the initial questionnaire was integrated by a team of physiotherapists and translators. Two translators majored in English and translated the initial sC-LKQ back to English, respectively. The back-translated questionnaires were compared with the original LKQ to ensure that there was no ambiguity in the Chinese version. The initial sC-LKQ was again modified according to the results of the comparison and language habits of Chinese. After evaluation and revision by a team of experts, the pilot test sC-LKQ was obtained.

Thirty participants aged over 18 years participated in the pilot test of the sC-LKQ. All the respondents were able to understand the meaning of each item and complete the questionnaire. The final version of the sC-LKQ was generated.

### 2.3. Instruments

For questionnaire validation, the original author of LKQ recommended comparing the RMDQ with the translated LKQ as a measure of construct validity (12). Two LBP-specific questionnaires and a demographic questionnaire created by our team made up this investigation. All participants performed the same pattern of completing basic demographic questions, sC-LKQ, and RMDQ on the Credamo platform.

#### 2.3.1. The Low Back Pain Knowledge questionnaire (LKQ)

The original LKQ consists of 16 questions in three dimensions: general knowledge (Q1, Q6, Q7, Q8, Q15), concepts (Q2, Q3, Q4, Q5), and treatment (Q9, Q10, Q11, Q12, Q13, Q14, Q16) of LBP, for a total of 24 points. It comprises eight single-choice and eight double-choice questions. Each question has five options, with one point indicating the correct answer. A higher score implies higher knowledge about LBP.

#### 2.3.2. The Roland-Morris Disability questionnaire (RMDQ)

In 1983, Roland and Morris developed the earliest RMDQ from the Sickness Impact Profile to a 24-item self-administration questionnaire, especially for back pain (22). It scores ranging from 0 (without any disability) to 24 (maximum disability) to evaluate the impact of pain during daily life. The simplified Chinese version of the RMDQ is reliable and valid as an LBP self-reported measurement tool in Mainland China (23).

### 2.4. Data analyze

Microsoft Office Excel 2019 was used for data organization. Further statistical analysis was conducted using IBM SPSS 28.0 (SPSS Inc., Chicago, United States). Scores on demographic indicators and items in the questionnaires were analyzed using descriptive statistics with expressed mean values and standard deviation (SD). Correlation analysis was performed to compare the association between demographic characteristics and sC-LKQ. A value of p of 0.05 or lower was regarded as statistically significant.

Cronbach’s alpha coefficient value was used to measure the internal consistency, and an alpha value higher than 0.70 indicated an acceptable internal consistency (24). The intraclass correlation (ICC) and Bland–Altman graph with 95% bound of the agreement were used to evaluate test–retest reliability. ICC value less than 0.5, between 0.5 and 0.75, and between 0.75 and 0.9 was considered poor, moderate and good test–retest reliability, respectively (25).

To assess the construct validity of the sC-LKQ through an exploratory factor analysis by the principal component with varimax rotation. The Kaiser-Meyer-Olkin (KMO) test was used to measure sampling adequacy of 0.6, and Bartlett’s test of sphericity significance level 0.05 was performed to establish the data sufficiency for structure identification and adequacy for principal component analysis (26).

Groups 1 and 2 (Chinese in China) were analyzed for differences with Chinese in Hungary, represented by Groups 5 and 6, using the Mann–Whitney U test. The significance level was set at *p* < 0.05.

## 3. Results

Of the 428 Chinese participants (183 males, 245 females), the mean age was 30.90 ± 11.30 years old. The demographic characteristics are illustrated in Table 1. The score of sC-LKQ was 14.25 ± 4.42. In the specific classification of the three blocks in sC-LKQ, the score of general knowledge was 5.45 ± 1.71 (total 9), the concept was 2.17 ± 1.13 (total 4), and the treatment was 6.62 ± 2.35 (total 11). A total of 137 participants had manifestations of LBP in the last 24 h at the time of testing (RMDQ score higher than 0). The scores in the six groups in the study are shown in Table 2. There were 264 participants without a medical education background who got 12.87 ± 4.53 points in sC-LKQ. Of these, the general knowledge part scored 4.98 ± 1.80, concepts scored 1.86 ± 1.06, and treatment scored 6.03 ± 2.43. Other 164 participants with medical education background got 16.46 ± 3.16 points in total and got 6.21 ± 1.22, 2.68 ± 1.05, and 7.57 ± 1.85 points in three sessions separately.

**Table 1:** Demographic characteristic of participants.

Variable	Mean (SD) or *N* (%)
Age (Ys)	30.895 (11.297)
Gender	
Male	183 (42.8)
Female	245 (57.2)
Education level	
Primary school	5 (1.2)
Middle school	14 (3.3)
High school	34 (7.9)
College	68 (15.9)
Bachelor degree	234 (54.7)
Master degree	64 (15.0)
P.hD. degree	9 (2.1)
Medical education background	
Yes	164 (38.3)
No	264 (61.7)

**Table 2:** Scores of the different subcategories of sC-LKQ.

	Number	sC-LKQ score	General knowledge	Concepts	Treatment
Group 1	66	14.83 ± 2.92	5.77 ± 1.23	2.12 ± 0.83	6.94 ± 1.74
Group 2	78	16.95 ± 3.05	6.21 ± 1.22	2.79 ± 1.01	7.95 ± 1.69
Group 3	61	12.33 ± 5.05	4.56 ± 1.98	1.92 ± 1.16	5.85 ± 2.76
Group 4	64	14.83 ± 3.00	5.77 ± 1.24	2.14 ± 0.81	6.92 ± 1.79
Group 5	64	16.50 ± 2.77	6.37 ± 1.18	2.66 ± 1.03	7.47 ± 1.64
Group 6	95	10.94 ± 4.93	4.36 ± 1.91	1.56 ± 1.24	5.01 ± 2.60
All	428	14.25 ± 4.42	5.45 ± 1.71	2.17 ± 1.13	6.62 ± 2.35

### 3.1. Internal consistency and test–retest reliability

**Figure 2:**
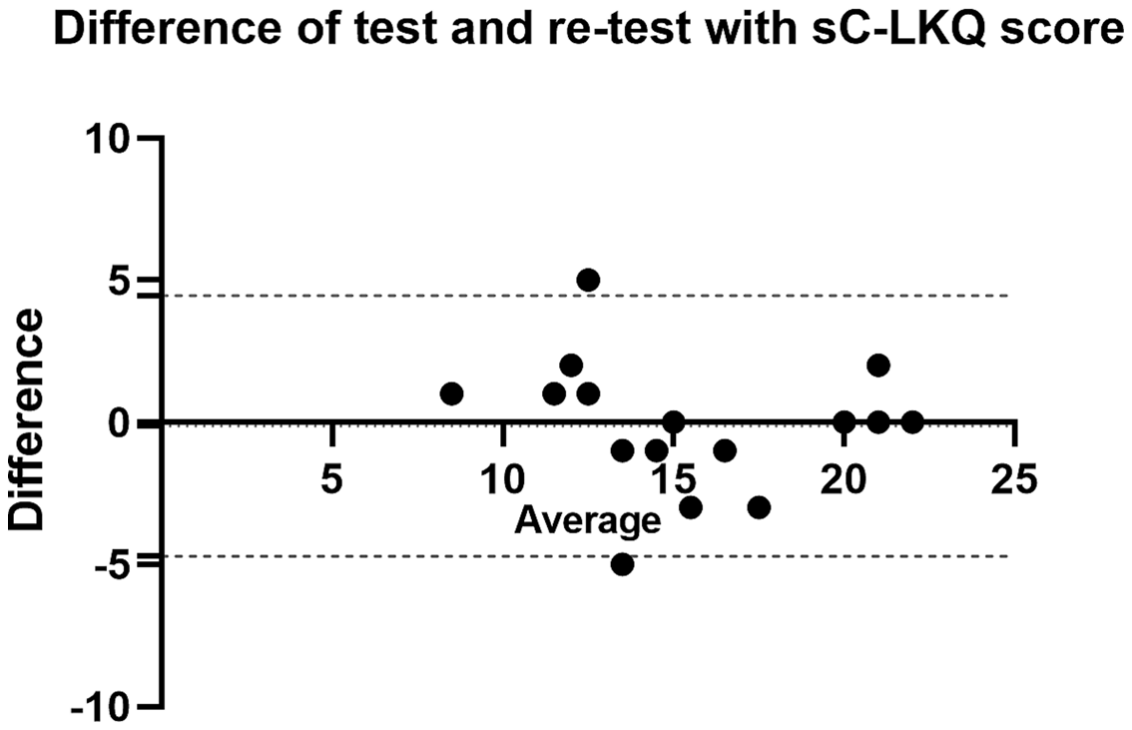
Bland–Altman plot of sC-LKQ score between test and retest. The solid black line indicates a different mean and the dashed black lines indicate a 95% agreement limit.

The sC-LKQ showed acceptable internal consistency, the Cronbach’s alpha coefficient was 0.79. The inter-item correlation counted based on the three dimensions from the original LKQ were 0.47 in general knowledge, 0.49 in concepts, and 0.66 in treatment. The ICC value was 0.85 (95% CI, 0.61–0.94), reflecting good test–retest reliability of sC-LKQ. The Bland–Altman plot graph is shown in Figure 2, with a mean value of −0.13 ± 2.34 (95% limits of agreement, −4.70 to 4.45). There was no significant proportional. bias between the test and retest.

### 3.2. Construct validity and concurrent validity

The KMO value was 0.864, and Bartlett’s test value of 1225.442 (*p* < 0.0001) indicated that the data were suitable for factor analysis. There were five components with eigenvalues greater than 1, occupying 53.67% of the cumulative rotation sums of squared loadings. The items showed factorial loads that varied from 0.321 to 0.835 (Table 3).

**Table 3:** The principal component analysis of sC-LKQ.

Items	Component					Communalities
	1	2	3	4	5	
Q1	**0.531**	−0.008	−0.113	**0.501**	−0.039	0.548
Q2	0.222	−0.007	−0.237	−0.274	**0.809**	0.835
Q3	**0.598**	−0.193	−0.164	0.073	0.041	0.428
Q4	**0.625**	−0.224	−0.203	0.120	−0.176	0.528
Q5	**0.457**	0.122	0.019	**0.462**	0.070	0.443
Q6	0.247	−0.154	**0.748**	0.305	0.210	0.781
Q7	**0.463**	−0.204	**0.426**	−0.122	0.186	0.486
Q8	0.230	**0.758**	0.058	0.126	0.101	0.657
Q9	**0.467**	−0.259	−0.014	−0.321	−0.248	0.450
Q10	0.384	**0.578**	0.152	−0.277	−0.209	0.625
Q11	**0.523**	0.088	0.315	−0.339	−0.138	0.515
Q12	**0.505**	0.249	−0.217	0.017	0.220	0.413
Q13	**0.522**	0.146	−0.081	−0.015	−0.212	0.346
Q14	**0.507**	−0.073	−0.196	0.111	−0.087	0.321
Q15	**0.793**	−0.167	−0.054	−0.139	0.005	0.678
Q16	**0.715**	−0.023	0.017	−0.136	0.059	0.533

The bold part indicates that the absolute value of the component load factor is greater than 0.4, indicating that the analysis item belongs to the corresponding component.

In the correlation analysis, RMDQ was found to be significantly and negatively correlated with the sC-LKQ score (*r* = −0.121, *p* = 0.012), level of education (*r* = −0.201, *p* < 0.001), and those without a medical education background (*r* = −0.097, *p* = 0.046). Macroscopically, the sC-LKQ score was statistically positively correlated with the level of education (*r* = 0.102, *p* = 0.035) and medical background (*r* = 0.407, *p* < 0.001). In terms of the coverage of the three modules of the sC-LKQ, the RMDQ was negatively and significantly correlated with scores in the category of general knowledge (*r* = −0.174, *p* < 0.001). Age had no statistically significant effect on the sC-LKQ and RMDQ (Table 4).

**Table 4:** Correlation analysis of sC-LKQ, RMDQ and demographical factors.

	sC-LKQ	RMDQ	Age	Education level	Medical background	General knowledge	Concepts
RMDQ	−0.121^*^						
Age	−0.078	0.056					
Education level	0.102^*^	−0.201^**^	−0.121^*^				
Medical background	0.407^**^	−0.097^*^	−0.299^**^	0.047			
General knowledge	0.831^**^	−0.174^**^	−0.078	0.105^*^	0.352^**^		
Concepts	0.723^**^	−0.088	−0.062	0.006	0.369^**^	0.502^**^	
Treatment	0.889^**^	−0.072	−0.040	0.110^*^	0.336^**^	0.584^**^	0.495^**^

**. Correlation (r) is significant at the 0.01 level (2-tailed).

*. Correlation (r) is significant at the 0.05 level (2-tailed).

### 3.3. Differences between Chinese in China and Hungary

There were 144 healthy Chinese participants in China and 159 in Hungary. After the Mann–Whitney U test, a significant statistical difference existed between Chinese people in China and Hungary (*p* < 0.001) in the sC-LKQ score. Chinese in China (15.98 ± 3.16) had higher sC-LKQ scores than Chinese in Hungary (13.18 ± 5.00).

## 4. Discussion

Self-efficacy is an important factor affecting chronic diseases. Patient knowledge is an essential component of primary prevention (27). Clinical practitioners in many countries have focused on the application and impact of LBP knowledge within the framework of their culture and have developed or validated scales to measure LBP knowledge (10–12, 28). However, there is a lack of validation for the Chinese LKQ. The purpose of this study was to complete the cross-cultural adaptation and reliability validation of the sC-LKQ to determine the characteristics of the scores in participants’ feedback.

The final version of the sC-LKQ was obtained after strict adherence to the steps of the Beaton cross-cultural study and pretesting to accomplish the trans-cultural adaptation of the LKQ (21). The demographic characteristics, sC-LKQ, and RMDQ were assessed in 428 participants. The sC-LKQ showed acceptable internal consistency (Cronbach’s alpha =0.783) among 16 items. It is higher than the result of the original English questionnaire (Cronbach’s alpha =0.71) (10) but lower than that of the Hungarian (Cronbach’s alpha =0.894) and one of the Arabic (Cronbach’s alpha =0.834) versions (11, 12). In another study verified by Jordanian scholars in the Arabic version of LKQ in 2021, Cronbach’s alpha was 0.707 (28). Notably, in a previous cross-sectional study performed by Chinese researchers, they derived a Cronbach’s alpha score for the LKQ that was almost identical to ours at 0.79 (29). Although Cronbach’s alpha values were slightly different across languages, the LKQ had high internal consistency in all existing validation studies from a statistical point of view. For test–retest reliability, the current study obtained an ICC of 0.847, which is similar to the results of 0.8–0.94 in the initial English LKQ (10). Therefore, the sC-LKQ has high reliability.

The construct validity results showed that the sC-LKQ could be divided into five components instead of the three aspects in the English version (10). Similar results also showed on the inter-item analysis; the three Cronbach’s alpha were not high according to the original three dimensions. A component analysis of the 16 questions revealed overlapping parts in some topics. According to the results, each of the five categories can be named as follows: specialty medical initiative (Q1-5, Q7, Q9, Q11-16), self-processing methods (Q8, Q10), disease manifestation (Q6, Q7), anatomical knowledge and identification (Q1, Q5), and precise LBP definition (Q2). The classification of the questions into four categories was obtained in a previous study (28). The influences that lead to these different categorization methods mostly come from differences in cultural and environmental backgrounds. Thus, the sC-LKQ is a comprehensive multidimensional questionnaire that promotes and improves patients’ limited health literacy and health outcomes through improved education and communication strategies (30, 31).

In this study, the average score of sC-LKQ was 14.25 ± 4.42. The scores for the three areas of general knowledge, concepts, and treatment each were 5.45 ± 1.71, 2.17 ± 1.13, and 6.62 ± 2.35, respectively. This result is similar to that of the previous Chinese LKQ study. The LKQ score was 14.82 ± 4.59 in total, 5.73 ± 1.84 in general knowledge, 2.18 ± 1.23 in concepts, and 6.92 ± 2.28 in treatment (29). These results corroborate that Chinese people have a low level of knowledge of the concept of LBP. It is worth noting that the participants of the previous study in China were all patients with LBP. In the present study, the LKQ score of LBP patients was 12.33 ± 5.00. The scores of the three corresponding knowledge were 4.56 ± 1.96, 1.92 ± 1.15, and 5.85 ± 2.73, respectively. From this perspective, the LKQ scores of patients with LBP in this study were lower than those reported in a previous Chinese study. The reason for this result might be that, in the previous study, the participants were all patients with LBP in tertiary care hospitals in Guangdong Province. People with such medical resources are in the top economic environment and education in China (32). On the contrary, our study did not set a geographic range for the participant population, which is more reflective of the knowledge of Chinese patients with LBP.

The sC-LKQ has acceptable concurrent validity by a strong connection with RMDQ. Spearman’s rank correlation coefficient showed that there were significant negative correlations between the sC-LKQ and the RMDQ. This finding was also reported by Kovács-Babócsay et al. (12) who indicated that the poorer the knowledge of spinal health, the more spinal problems occur. Meanwhile, the sC-LKQ score had a significant positive correlation with education level and medical background. This also accords with our earlier observations, which showed that people living in places with superior educational resources have a higher level of knowledge about a specific disease. It is important to note that among the results of correlation analyses, the results were shown weak correlation, except between the sC-LKQ and medical background. This was also presented in previous research (12), and one of the possibilities is the increase in individual differences due to the large sample size.

Prior studies have also focused on the knowledge of healthcare professionals about LBP (Table 5) (10, 12, 28, 33). However, the findings from the current study of sC-LKQ in individuals with medical education backgrounds got lower scores. There are several possible explanations for this finding. First, except for the nurses in the study from Kanaan, all other previous studies selected medical personnel closely associated with LBP, such as physical therapists (28). In our study, not all specialize in spinal health or related fields. It is also reported from Kanaan’s study that there were differences in the knowledge of LBP among medical professionals with different orientations (28). Another possible explanation for this is the differences in sample size. In previous studies, it ranged from 20 to 60, whereas the number of participants in this category in the current study was 164. A larger sample of participants is reflective of the characteristics of the group in a specific setting.

**Table 5:** The LKQ scores in previous studies among healthcare professionals.

Author (year)		LKQ score	General knowledge	Concepts	Treatment
Maciel et al. (2009)		23.55 ± 0.60	8.85 ± 0.36	3.90 ± 0.30	10.80 ± 0.41
Morimoto et al. (2018)		19.1 ± 2.5	8.0 ± 0.8	3.1 ± 0.9	8.0 ± 1.7
Kovács-Babócsay et al. (2019)		19.1	7.8	3.4	7.9
Kanaan et al. (2021)	Physical therapists	16.80 ± 2.38	7.05 ± 1.23	2.95 ± 0.83	6.80 ± 1.40
	Nurses	10.85	4.40	1.95	4.50

It is interesting to note that the sC-LKQ scores differed between the Chinese in China and Hungary. The variation in this result is mainly attributed to the differing demographics. Individuals with and without a medical background were included in the analysis. The participants in Hungary were mostly local Chinese students who were studying there; their overall age was younger, and they lacked LBP knowledge.

This study has several limitations. Although the selection of most participants in China in this study was not geographically limited, the study’s participants with LBP in China were primarily from Sichuan Province and were not fully representative of the entire Chinese population. In addition, the questionnaires were completed online; therefore, errors due to the participants during the filling process could not be avoided.

## 5. Conclusion

The current study showed that the sC-LKQ has sound reliability and validity. It can be used in clinical practice to evaluate the self-efficacy of patients with LBP. In addition, it can be used as a valid evaluation tool in Chinese research on LBP.

## Data availability statement

The raw data supporting the conclusions of this article will be made available by the authors, without undue reservation.

## Ethics statement

The studies involving humans were approved by Local Ethics Committee of Chengdu Sports University Hospital and the Institutional Review Board of the Regional Research Committee of Clinical Center of University of Pecs. The studies were conducted in accordance with the local legislation and institutional requirements. The participants provided their written informed consent to participate in this study.

## Author contributions

MJ and AM planned the original idea and designed the study. ZW and YX collected the data. AM, ZW, and YX performed the data analysis. ZW contributed to drafting this article. All authors contributed to the article and approved the submitted version.

## Abbreviations

LBP: low back pain
LKQ: low back pain knowledge questionnaire
sC-LKQ: simplified Chinese Low Back Pain Knowledge questionnaire
RMDQ: Roland-Morris Disability Questionnaire
SD: standard deviation
ICC: intraclass correlation
KMO: Kaiser-Meyer-Olkin
